# Multiple Mycetoma Lung Secondaries from Knee Eumycetoma: An Unusual Complication

**DOI:** 10.1371/journal.pntd.0004735

**Published:** 2016-07-21

**Authors:** EL Samani Wadaa Mohamed, Nancy Seif EL Din, Ahmed Hassan Fahal

**Affiliations:** Mycetoma Research Centre, University of Khartoum, Khartoum, Sudan; University of California San Diego School of Medicine, UNITED STATES

## Case Presentation

The reported patient was a 25-year-old university student from the White Nile State in central Sudan who had right knee eumycetoma caused by *Madurella mycetomatis*. His condition started in 2002 with a small, painless knee swelling, which gradually increased in size. A year later, he developed one sinus and started to discharge sero-purulent discharge containing black grains. He was seen at a primary health care center, but neither a diagnosis was established nor a treatment was advised.

In 2009, he presented to the Mycetoma Research Centre (MRC), University of Khartoum, Khartoum, Sudan, and the diagnosis of right knee *M*. *mycetomatis* eumycetoma was established by cytological examination of the lesion fine needle aspirates. The disease extent was determined by the knee X-ray, ultrasound, and MRI examinations that showed he had massive soft tissue involvement but no bone affection. The patient was commenced on Ketoconazole 800 mg daily; however, three months later, he discontinued the treatment due to his frustrations with the slow improvement, the high costs of treatment, and the chronicity of the condition. The lesion continued its aggressive course with progressive increase in size and the development of multiple sinuses.

In 2012, he underwent wide local surgical excision and skin grafting with uneventful post-operative recovery, and 800 mg Voriconazole daily was commenced for 18 months. He, again, was unable to continue on treatment due to the unavailability of the drug. This resulted in the development of massive recurrence and satellite lesion in the right inguinal region, containing multiple discharging sinuses. In 2013, he underwent further wide local surgical excision for the knee and the inguinal satellite and was started on Itraconazole 800 mg daily, but he discontinued the treatment and follow-up due to his low socioeconomic status and financial constraints.

He was seen in 2014 with severe fixed flexion deformity of knee and pain due to secondary bacterial infection. The pain was controlled with analgesia. In June 2015, he presented again to the MRC with general weakness, severe weight loss, chronic persistent cough, knee pain, and walking disability.

**Fig 1:**
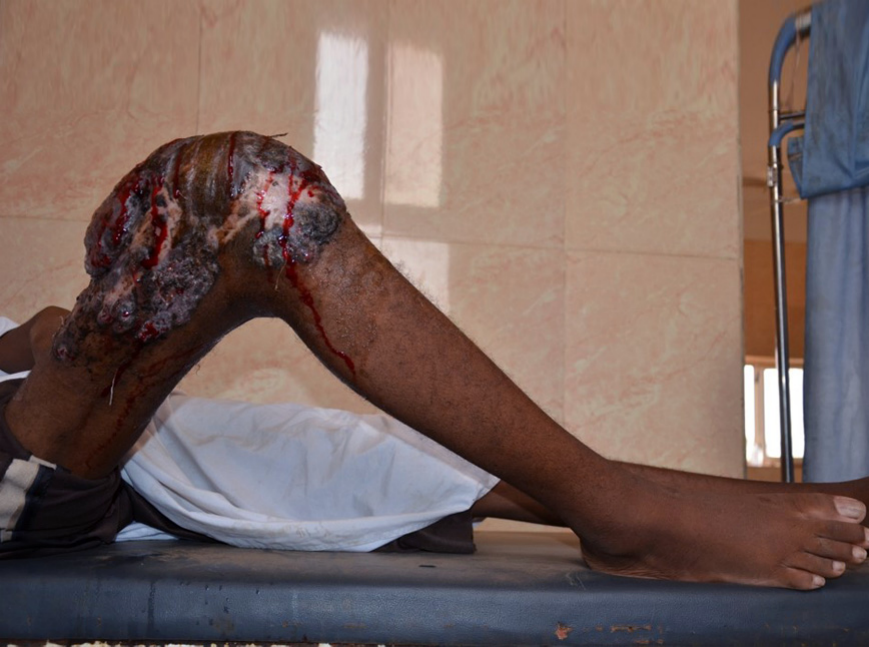
Showing massive knee eumycetoma with multiple active sinuses, discharge, and black grains.

On examination, he was pale, cachexic, and extremely ill. He was suffering from depression. Systemic examinations were unremarkable apart from fingers clubbing, bilateral basal crepitations, and bronchial breathing. Local examination showed fixed flexion deformity of knee and a massive mycetoma lesion, involving the whole knee region extending to the leg and thigh with multiple sinuses and sero-purulent discharge containing numerous black grains. There was a huge right inguinal region satellite with multiple non-discharging sinuses. All sensations and peripheral pulses in the right lower limb were normal, however, there was pitting edema (Fig 1).

His hepatic and renal profiles were normal. The hematological profile showed microcytic hypochromic anemia with hemoglobin of 5 g/L. Bone marrow biopsy was performed, and it revealed normal reactive bone marrow feature. Sputum for acid-alcohol fast bacilli was negative.

**Fig 2:**
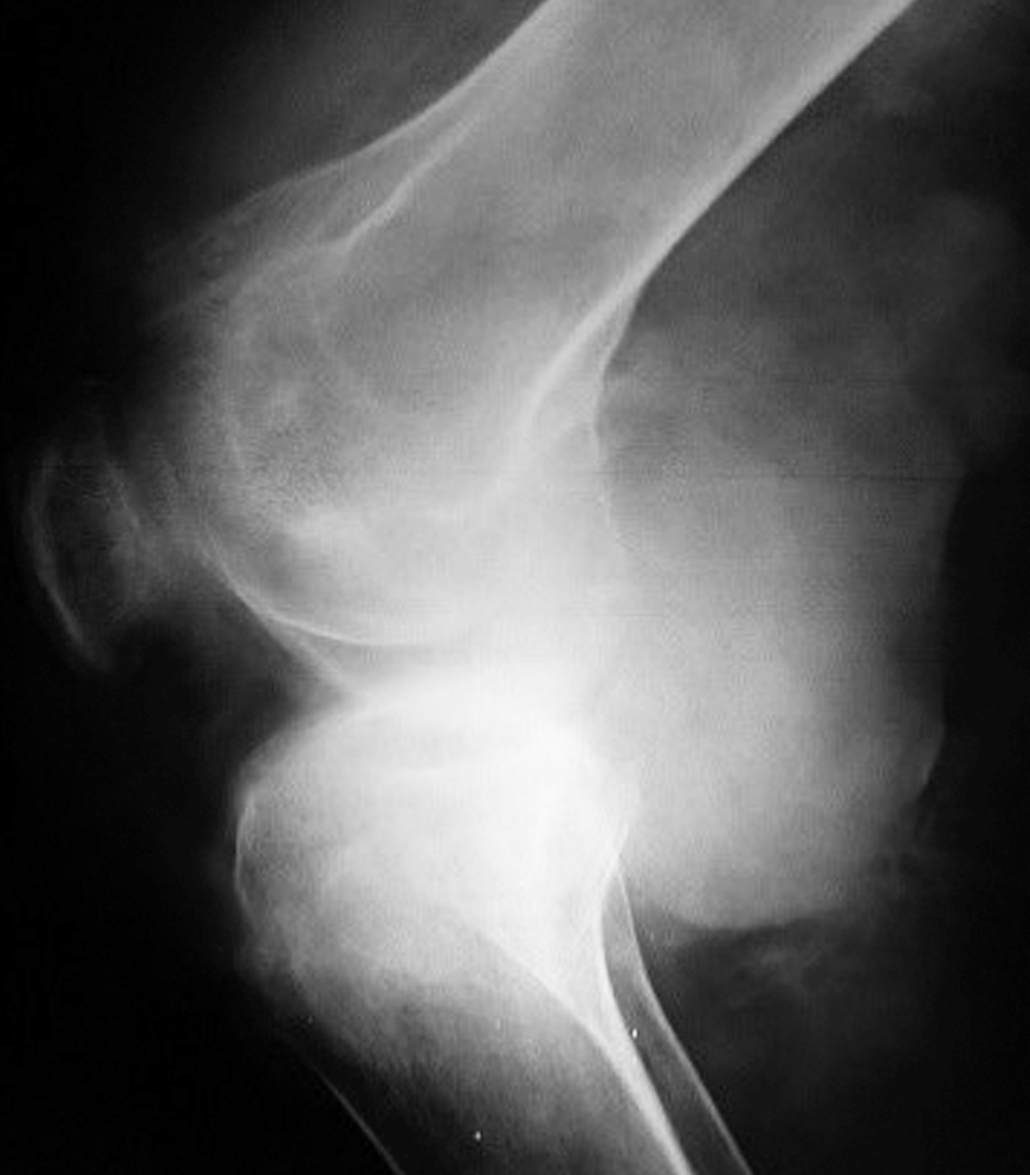
X-ray of the patient’s right knee joint region showing massive soft tissue mass and condylar notch widening with massive effusion.

**Fig 3:**
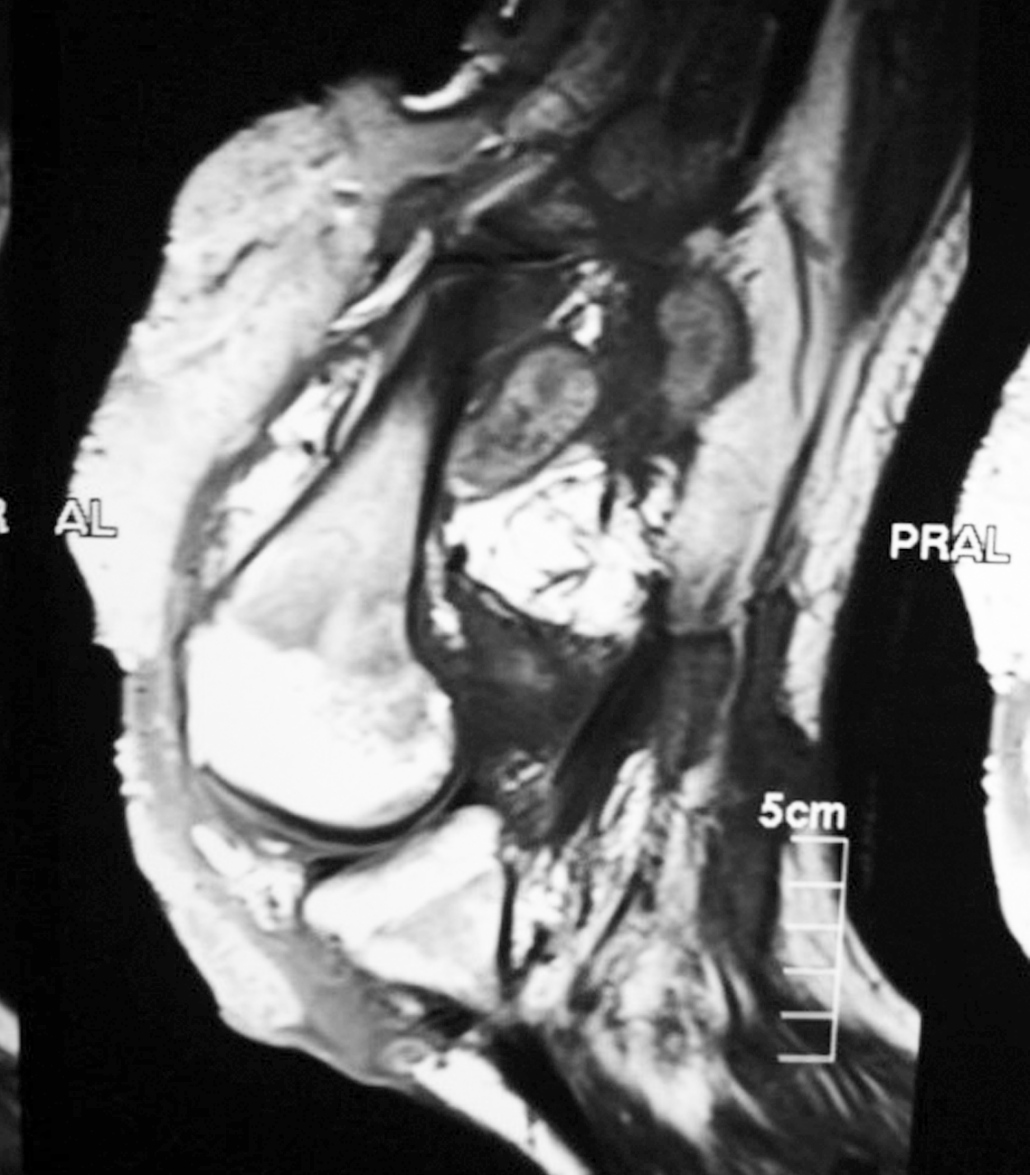
The knee region MRI showing extensive enhancing solid mass with nodule components, infiltrating the muscular components of anterior and posterior lower thigh with intra-articular extension and bony cortical defect at lateral femoral condyle.

**Fig 4:**
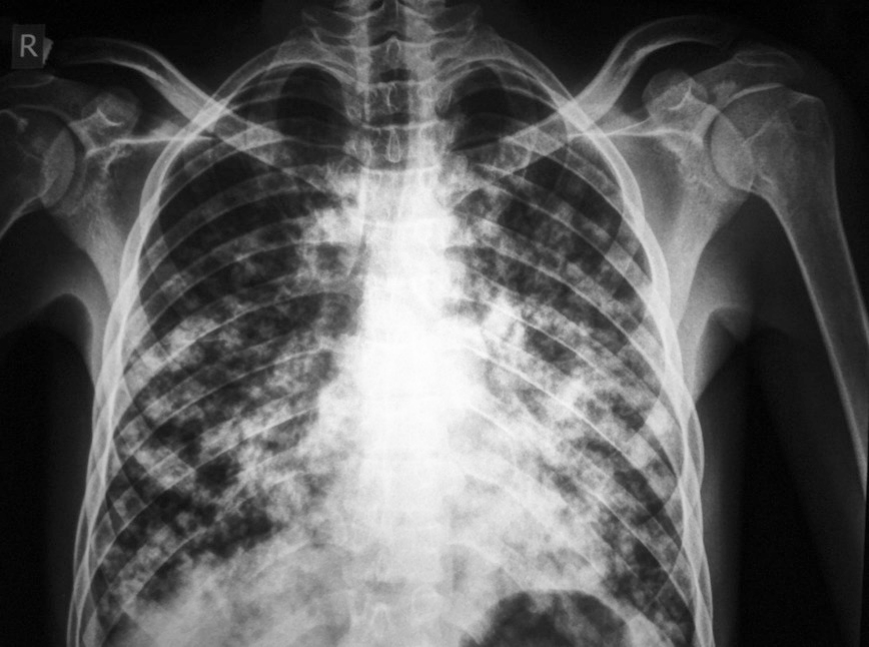
Chest X-ray showing multiple bilateral lungs patches with the differential diagnosis of secondaries, miliary tuberculosis, or mycetoma infiltrations.

**Fig 5:**
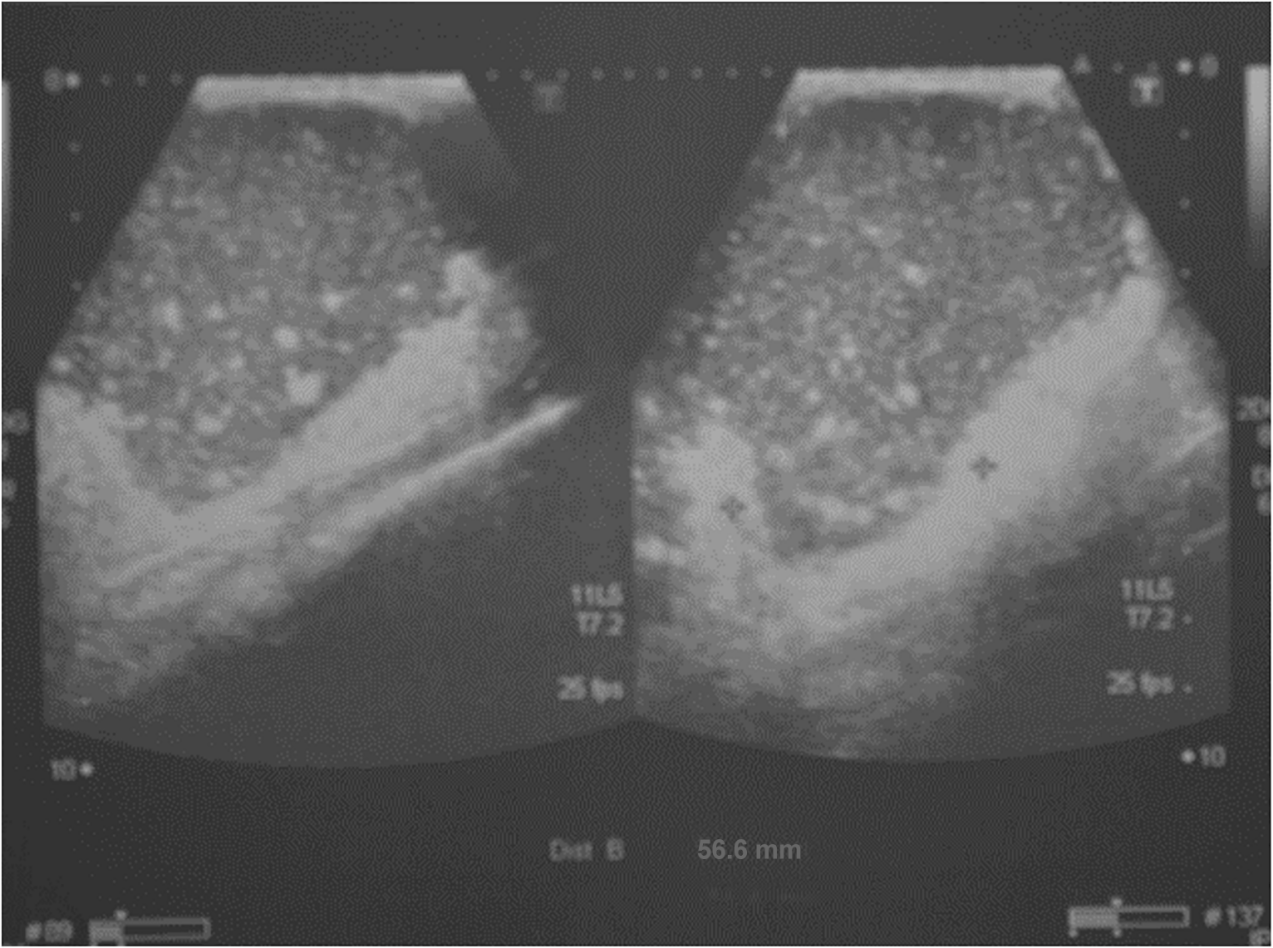
Chest CT scan showing numerous multiple lung nodules with multiple dot-in sign characteristic for mycetoma.

The knee joint X-ray showed a massive soft tissue mass with condylar notch widening and massive effusion (Fig 2). The knee region MRI showed extensive enhancing solid mass with nodule components, infiltrating the muscular components of anterior and posterior lower thigh with intra-articular extension and bony cortical defect at lateral femoral condyle (Fig 3). The chest X-ray revealed multiple bilateral lungs patches with the differential diagnosis of secondaries, miliary tuberculosis, or mycetoma infiltrations (Fig 4). He had a chest CT scan, which showed numerous multiple lung nodules, highly suggestive of lung mycetoma (Fig 5). A chest MRI was done and confirmed the CT scan findings. Grains obtained from early morning sputum were cultured and showed evidence of *M*. *mycetomatis*. Further confirmation was obtained by PCR and LAMP molecular diagnostic techniques.

**Fig 6:**
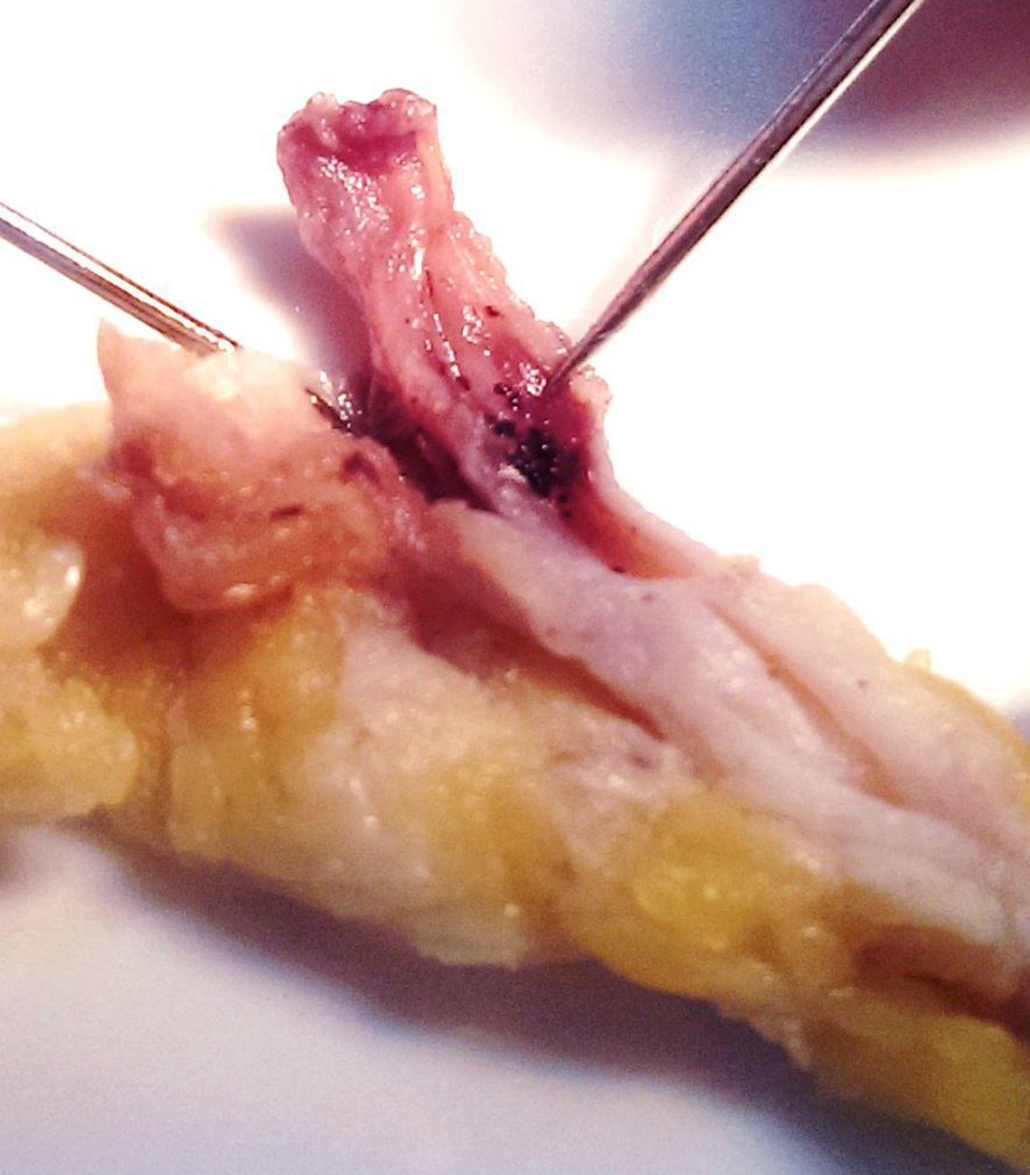
The thigh mass ultrasound examination showing multiple massive cystic masses with hyper-reflective echoes in line with eumycetoma.

The patient received several blood transfusions, antibiotics for the secondary bacterial infection, nutritional support, and he underwent above-knee amputation and wide excision of the inguinal satellite. During surgery, the great saphenous vein was found to be studded with black grains. He had uneventful post-operative recovery. Sadly, later on, he developed a massive cystic lesion in the left mid-thigh. Ultrasound examination revealed multiple massive cystic masses with hyper-reflective echoes in line with eumycetoma (Fig 6). Cytopathological smears revealed *M*. *mycetomatis* grains with inflammatory infiltrates. His general condition continued to deteriorate, and he died from massive lung disease and sepsis.

## Case Discussion

Mycetoma is a serious neglected medical and health problem with a worldwide distribution, though endemic in many tropical and subtropical regions [1,2]. It is caused by certain true fungi (eumycetoma) and bacteria (actinomycetoma), leading to chronic granulomatous inflammatory disease, characterized by deformity, destruction, and disability [3,4]. Mycetoma is basically a localized subcutaneous disease that spreads locally along the fascial planes to affect the skin, deep structures, and bone [5]. Rarely, it can spread along the lymphatics to the regional lymph node to produce regional satellite [6]. Few blood-spread mycetoma cases were reported [7]. In mycetoma, generalized constitutional symptoms are rare, and that may include anemia, weight loss, general weakness, and depression. They are seen in patients with long-standing enormous disease, with severe secondary bacterial infection of the open sinuses, malnutrition, and septic bone morrow depression [8]. These symptoms were clearly documented in the reported patient in this communication.

To our knowledge, eumycetoma lung spread and secondary infiltration were not previously reported. The mechanism that produces this phenomenon is unclear; however, the fact that the great saphenous veins were studded with numerous black grains and the development of a cystic eumycetoma lesion in the right thigh may support the blood-spread theory.

The aggressive mycetoma behavior documented here is unique and unreported. The interrupted treatment observed in this patient may be an important factor in the mycetoma aggressive course. The causes of the treatment interruption were multifactorial and included the disease’s painless nature, patient’s low socioeconomic status, poor health education, the unavailability of the prescribed medicines, and the absence of medical facilities in his remote village. The patient’s depressed immune system or the aggressive nature of the causative organism may have contributed to this presentation.

The lung secondaries seen in the reported patient are different from the pulmonary mycetoma. The latter has a distinctive clinical–radiological presentation. It is due to colonization of aspergillus or candida species in pre-existing pulmonary cavities as a consequence of different diseases, including lung tuberculosis and abscess, bronchiectasis, bullous emphysema commonly seen in patients with leukemia, lymphoma, diabetes mellitus, and patients on corticosteroids, immunosuppressive drugs, or chemotherapy [9]. Cough and hemoptysis are the typical features of pulmonary mycetoma, and these patients have a typical chest X-ray appearance. The diagnosis of pulmonary mycetoma is confirmed by positive sputum culture for aspergillus or candida species and a positive aspergillus precipitin test. These patients respond to various antifungal drugs [10]. All these features were not seen in the reported patient.

## Ethical Statement

The patient’s informed consent was obtained for publication. This report was approved by the Mycetoma Research Centre Institutional Review Board.

**Key Learning Points**

- Mycetoma is a localized disease, yet rarely, it can spread to distant organs.
- Lung spread is a scarce occurrence in mycetoma.
- In some patients, *Madurella mycetomatis* can progress widely without response to the different treatment modalities.
- Vascular spread, which is a rare phenomenon in mycetoma, may explain the lung spread encountered in this patient.
- Treatment compliance is essential for good clinical outcome.
